# The Association Between Location of BRCA Mutation and Efficacy of PARP Inhibitor as a Frontline Maintenance Therapy in Advanced Epithelial Ovarian Cancer

**DOI:** 10.3390/cancers17050756

**Published:** 2025-02-23

**Authors:** Ji Hyun Kim, Se Ik Kim, Eun Taeg Kim, Hyeong In Ha, Dong-eun Lee, Yong Jae Lee, Jung-Yun Lee, Sunghoon Kim, Sang Wun Kim, Young Tae Kim, Sang-Yoon Park, Myong Cheol Lim, Eun-Ji Nam

**Affiliations:** 1 Center for Gynecologic Cancer, National Cancer Center, Goyang 10408, Republic of Korea; jihyunkim@ncc.re.kr (J.H.K.); parksang@ncc.re.kr (S.-Y.P.); 2 Department of Obstetrics and Gynecology, Institute of Women’s Medical Life Science, Severance Hospital, Yonsei University College of Medicine, Seoul 03722, Republic of Korea; svass@yuhs.ac (Y.J.L.); jungyunlee@yuhs.ac (J.-Y.L.); shkim70@yuhs.ac (S.K.); san1@yuhs.ac (S.W.K.); ytkchoi@yuhs.ac (Y.T.K.); 3 Department of Obstetrics and Gynecology, Seoul National University College of Medicine, Seoul 03080, Republic of Korea; seikky@naver.com; 4 Department of Obstetrics and Gynecology, Kosin University College of Medicine, Pusan 49267, Republic of Korea; dikei03@naver.com; 5 Department of Obstetrics and Gynecology, Pusan University College of Medicine, Yangsan 50612, Republic of Korea; hi126908111@gmail.com; 6 Biostatistics Collaboration Team, Research Core Center, National Cancer Center, Goyang 10408, Republic of Korea; dong-eun@ncc.re.kr; 7 Cancer Control and Policy, National Cancer Center Graduate School of Cancer Science and Policy, National Cancer Center, Goyang 10408, Republic of Korea; 8 Rare & Paediatric Cancer Branch and Immuno-Oncology Branch, Division of Rare and Refractory Cancer, Research Institute, National Cancer Center, Goyang 10408, Republic of Korea

**Keywords:** ovarian cancer, BRCA, PARP inhibitor

## Abstract

Background: The location of BRCA mutations within functional domains may affect sensitivity to poly (ADP-ribose) polymerase (PARP) inhibitors and platinum-based chemotherapy. This study aimed to evaluate the progression-free survival (PFS) benefit from the PARP inhibitor in relation to the location of mutations in *BRCA1*/*BRCA2* in newly diagnosed ovarian cancer. Materials and methods: Patients with advanced stage III-IV epithelial ovarian cancer who had deleterious *BRCA1* or *BRCA2* were analyzed. PFS and clinical and molecular data were compared between patients who received olaparib or niraparib as frontline maintenance therapy and those who did not. Subgroup analyses were conducted based on the location of *BRCA* mutations within the functional domain or the ovarian cancer cluster region (OCCR). Results: Of the 380 patients, 242 (63.7%) harbored *BRCA1* mutation, 137 (36.1%) harbored *BRCA2*, and one (0.3%) harbored both *BRCA1* and *BRCA2*. With a median follow-up of 35.8 months, the DNA binding domain in *BRCA1* (HR, 0.34; 95% CI, 0.15–0.79; *p* = 0.01) and *BRCA2* (HR, 0.25; 95% CI, 0.08–0.78; *p* = 0.01) demonstrated particularly significant benefit. In patients who harbored *BRCA1* mutation in the C-terminal domain (BRCT), no statistically significant PFS benefit from PARP inhibitor was observed (HR, 0.76; 95% CI, 0.39–1.52; *p* = 0.44). PFS benefit from PARP inhibitor maintenance was observed in both OCCR (HR, 0.49; 95% CI, 0.32–0.74; *p* < 0.01) and non-OCCR (HR, 0.51; 95% CI, 0.27–0.63; *p* < 0.01). Conclusions: Frontline PARP inhibitor maintenance therapy demonstrated a significant PFS benefit in patients with *BRCA1*/*2* mutations, with particularly pronounced benefits for those with mutations located in the DBD of *BRCA1* and *BRCA2*. However, the benefit was less evident for patients with *BRCA1* mutations located in the BRCT domain.

## Simple Summary

This study investigated how the location of *BRCA1*/*2* mutations affects the benefit of PARP inhibitor maintenance therapy in newly diagnosed advanced ovarian cancer. Among 380 patients, those with *BRCA1* or *BRCA2* mutations in the DNA binding domain (DBD) showed a significant progression-free survival (PFS) benefit from PARP inhibitors. In contrast, patients with *BRCA1* mutations in the C-terminal BRCT domain did not show a significant benefit. These findings highlight the importance of mutation location in predicting response to PARP inhibitor therapy.

## 1. Introduction

Ovarian cancer is the leading cause of death among gynecologic cancers, with approximately 324,400 new cases and 207,000 deaths reported in 2022 [1,2]. At the time of diagnosis, the majority of ovarian cancer patients present with advanced-stage disease characterized by peritoneal carcinomatosis. Although up to 80% of patients respond to frontline chemotherapy, approximately 75% experience relapse within a median of 18 to 24 months in the absence of maintenance therapy.

The introduction of Poly (ADP-ribose) polymerase (PARP) inhibitor as a maintenance therapy has led to major changes in the approaches to managing patients with *BRCA*-mutated newly diagnosed epithelial ovarian cancer [3,4,5]. In the pivotal SOLO1 trial, olaparib demonstrated a durable progression-free survival (PFS) benefit beyond the end of treatment in patients with advanced ovarian cancer and *BRCA1*/*2* mutations [3]. Similarly, in the PRIMA trial, niraparib significantly improved survival outcomes in patients with homologous recombination deficiency (HRD), including those with *BRCA1* or *BRCA2* mutations, in newly diagnosed advanced ovarian cancer at high risk of recurrence [4,6]. Both olaparib and niraparib have been approved for first-line maintenance treatment, with no significant difference in PFS or overall survival (OS) observed between the two agents [7].

*BRCA1* and *BRCA2* are two large genes, with exon 11 comprising a substantial portion of both [8,9]. These genes harbor distinct functional domains, which are specific regions within the proteins that facilitate DNA repair and maintain genome stability. *BRCA1* is characterized by three key functional domains: the N-terminal RING domain, a DNA-binding domain (DBD) essential for DNA repair, and the C-terminal BRCT domain, which binds phosphorylated proteins involved in the DNA damage response [10,11]. *BRCA2* has two key functional domains, which play a crucial role in homologous recombination by recruiting *RAD51* recombinase to double-strand breaks: a *RAD51*-binding domain (*RAD51*-BD), and a highly conserved C-terminal DBD [11].

Several studies have indicated that the location of *BRCA* mutations within functional domains may affect sensitivity to PARP inhibitors and platinum-based chemotherapy. For instance, a post hoc analysis of the PAOLA trial demonstrated that the PFS benefit of maintenance therapy with olaparib and bevacizumab was particularly notable in patients with mutations located in the DBD of *BRCA1* [12]. Building upon these findings, this study aimed to evaluate the impact of *BRCA1*/*2* mutation location on the PFS benefit conferred by maintenance therapy with PARP inhibitors.

## 2. Materials and Methods

### 2.1. Patients and Study Design

From July 2019 to December 2022, we enrolled patients who were newly diagnosed with epithelial ovarian cancer, fallopian tube carcinoma, or primary peritoneal carcinoma from four hospitals in Korea. This study was a multicenter retrospective analysis conducted in compliance with the Declaration of Helsinki, Good Clinical Practice guidelines, and all applicable local laws and regulations. Ethical approval was granted by the institutional review boards of the four participating centers in Korea: National Cancer Center (NCC2023-0024), Seoul National University Hospital (H-2108-169-1248), Severance Hospital (4-2024-0835), and Kosin University Hospital (KUGH 2023-03-008). The requirement for obtaining informed consent was waived.

Patients who met the following criteria were included in this study: (1) diagnosed with International Federation of Gynecology and Obstetrics (FIGO) stage III and IV disease; (2) completed at least four cycles of frontline platinum-based chemotherapy and achieved either a complete or partial response, as determined by investigators; and (3) carried deleterious germline or somatic mutations in *BRCA1* and/or *BRCA2* genes. BRCA testing was primarily performed using tumor specimens obtained during diagnostic surgery, ensuring molecular profiling from the primary tumor site. Exclusion criteria included patients with BRCA wild-type tumors or variants of unknown significance (VUS), those who used bevacizumab as a frontline maintenance treatment, those with insufficient clinical data, or those lost to follow-up during frontline treatment. Patients who received both bevacizumab and olaparib as a maintenance were excluded from this study.

### 2.2. Study Outcomes

The main objective of this study was to assess PFS between patients who received PARP inhibitors (niraparib or olaparib) as a frontline maintenance treatment, and those who did not. PFS was defined as the time from the completion of platinum-based chemotherapy to disease progression or death from any cause, whichever occurred first. Disease assessment was conducted by the investigators, using computed tomography or positron emission tomography-computed tomography scan every 3 to 6 months in accordance with Response Evaluation Criteria in Solid Tumors, version 1.1 [13].

PFS was assessed according to the location of the *BRCA1*/*2* mutation. The description of mutations was given at the genomic level on transcripts NM_007294.3 (*BRCA1*) and NM_000059.3 (*BRCA2*) on Human Genome hg19. The locations of *BRCA1*/*2* variants are grouped into functional domains and ovarian cancer cluster regions. For *BRCA1*, the functional domains were defined as follows: (i) RING domain: amino acids (AA) 8–96; DBD: AA 452–1092; BRCT: AA 1646–1736 and 1760–1855 [14]. For *BRCA2*, functional domains were defined as follows: (i) *RAD51*-BD: AA 900–2000; (ii) DBD: AA 2459–3190 [15].

The ovarian cancer cluster region (OCCR) was associated with a relative increase in ovarian cancer risk to breast cancer, compared to other regions. For *BRCA1*, OCCR is located from c.1380 to c.4062. For *BRCA2*, there are multiple OCCRs: c.3249 to c.5681, and c.6645 to c.7471 [16].

### 2.3. Statistical Analysis

Baseline characteristics, including age, histology, FIGO stage, timing of surgery, postoperative residual disease, CA-125, and response to platinum-based chemotherapy, are compared between patients who received PARP inhibitors and those who did not. For categorical variables, comparisons between patients treated with PARP inhibitors and those without were conducted using the chi-square test, Z-test, or Fisher’s exact test, where appropriate. Continuous paired data were analyzed using the Wilcoxon rank sum test. PFS was estimated using the Kaplan–Meier method, and survival differences between the groups were compared using the log-rank test.

All statistical analyses were conducted using R (version 4.2.1, R Foundation for Statistical Computing, Vienna, Austria) and SAS software (version 9.4 or later, SAS Institute Inc., Cary, NC, USA). A two-tailed *p*-value of less than 0.05 was considered statistically significant.

## 3. Results

### 3.1. Patient Characteristics

A total of 380 patients who harbored *BRCA1*/*2* mutations were included in the analysis. Of these, 209 (55.0%) patients received PARP inhibitor as a frontline maintenance therapy. In total, 168 patients (80.4%) received olaparib, and 41 patients (19.6%) received niraparib.

The clinical characteristics of the two groups are summarized in Table 1. Overall, the median age at diagnosis was 57 years (interquartile range [IQR]; 49–64). In total, 94.2% (358/380) of patients were high-grade serous carcinoma, and 44.5% (169/380) of patients were diagnosed at stage IV. Neoadjuvant chemotherapy followed by interval cytoreductive surgery was performed in 47.4% (180/380), and 64.3% (243/380) of patients achieved no gross residual disease after surgery. The median CA-125 level at initial diagnosis was 1080 (IQR; 381–3160). In total, 341 (89.7%) patients achieved a clinically complete response, which was defined as having no evidence of disease or complete response. All variables found no significant difference between patients who received frontline PARP inhibitor maintenance therapy than in those who did not.

### 3.2. Location and Type of Mutations in BRCA1 and BRCA2

Of the 380 patients, 242 (63.7%) harbored *BRCA1* pathologic or likely pathologic variants (PV/LPV), 137 (36.1%) harbored *BRCA2*, and one (0.3%) harbored both *BRCA1* and *BRCA2*. Mutational type and location of mutation are summarized in Table 2. Frameshift mutations were the most common in mutational type, observed in 46.4% (175/377) of cases, followed by missense mutations (34.0%, 128/377), nonsense mutations (9.3%, 35/377), splice-site mutations (6.6%, 25/377), and large rearrangements (3.7%, 14/377). No significant difference in mutational types was observed between the two groups. Regarding the cluster region, 49.1% (185/377) of mutations were located within the OCCR, while 50.9% (192/377) were located outside the OCCR.

For *BRCA1* variants (N = 240), most mutations were located in the BRCT domain (21.3%, 51/240), followed by the DNA binding domain (15.4%, 37/240), and the RING domain (3.8%, 9/240). For *BRCA2* variants (N = 137), 30.7% (42/137) occurred in the DBD, and 34.3% (47/137) in the *RAD51*-binding domain. The distribution of specific binding domain mutations did not differ significantly between the two groups for either *BRCA1* (*p* = 0.22) or *BRCA2* (*p* = 0.25).

### 3.3. Survival Outcome According to Location of Mutated Gene

**Figure 1:**
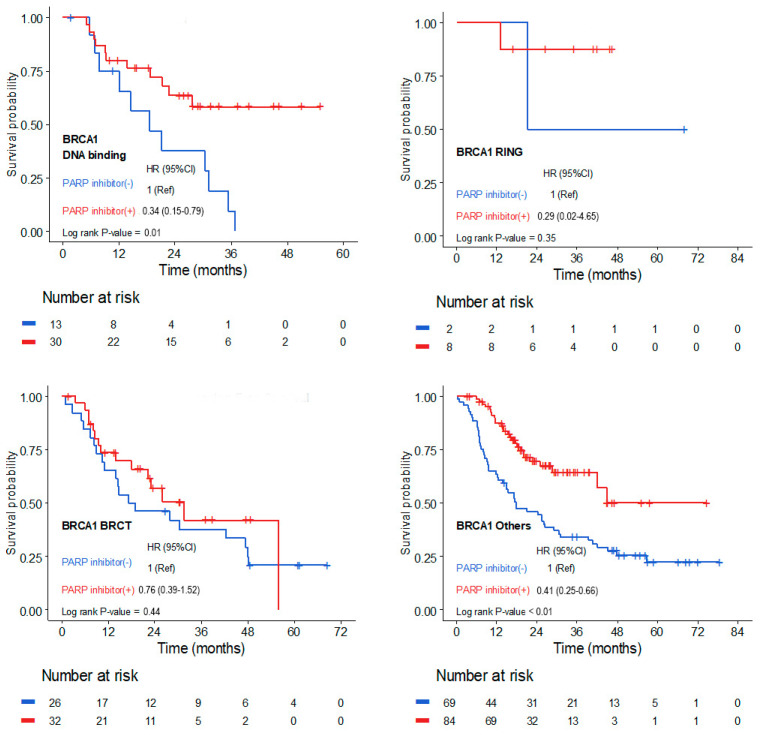
The Kaplan–Meier Curve for progression-free survival by mutation locations within the *BRCA1* domain.

**Figure 2:**
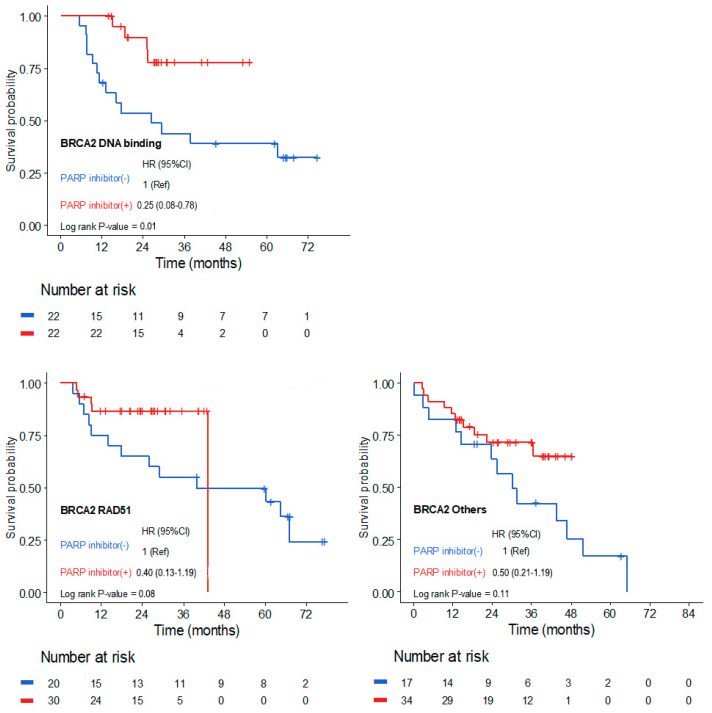
Kaplan–Meier Curve for progression-free survival by mutation locations within the *BRCA2* domain.

With a median follow-up of 35.8 months (IQR, 31.8–39.6), PFS outcomes varied among subgroups defined by mutation locations within *BRCA1*/*2* domains (Figure 1 and Figure 2). For *BRCA1*, patients with mutations in the DBD exhibited a significantly improved response to PARP inhibitor therapy, with a hazard ratio of 0.34 (95% CI, 0.15–0.79) compared to those not receiving PARP inhibitors (*p* = 0.01; Figure 1. In contrast, patients with *BRCA1* mutations in the BRCT domain showed a less pronounced benefit from PARP inhibitor therapy, with a hazard ratio of 0.76 (95% CI, 0.39–1.52; *p* = 0.44; Figure 1. For patients with *BRCA1* mutations located outside functional domains, PARP inhibitor therapy resulted in a significant improvement in PFS compared to no PARP inhibitor use, with a hazard ratio of 0.41 (95% CI, 0.25–0.66; log-rank *p* < 0.01; Figure 1).

For *BRCA2*, patients with mutations in the DBD demonstrated a significantly enhanced response to PARP inhibitor therapy, with a hazard ratio of 0.25 (95% CI, 0.08–0.78, *p* = 0.01; Figure 2). Similarly, patients with mutations in the *RAD51*-binding domain also demonstrated a substantial benefit from PARP inhibitors, with a hazard ratio of 0.40 (95% CI, 0.13–1.19; *p* = 0.08; Figure 2). In contrast, patients with *BRCA2* mutations located outside functional domains showed a less pronounced, statistically non-significant improvement in PFS, with a hazard ratio of 0.50 (95% CI, 0.21–1.19; *p* = 0.11; Figure 2).

**Figure 3:**
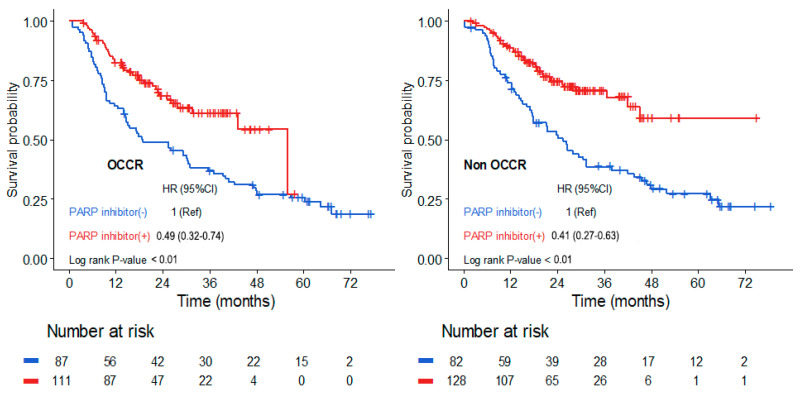
Kaplan–Meier Curve for progression-free survival by mutation locations within the ovarian cancer cluster region.

PFS outcomes were analyzed based on the presence or absence of *BRCA1*/*2* mutations in the OCCR (Figure 3). Patients with mutations located within the OCCR demonstrated a significant benefit from PARP inhibitor therapy, with a hazard ratio of 0.49 (95% CI, 0.32–0.74, *p* < 0.01; Figure 3. Similarly, patients with *BRCA* mutations outside the OCCR (non-OCCR) also experienced a substantial improvement in PFS with PARP inhibitors, with a hazard ratio of 0.41 (95% CI, 0.27–0.63; *p* < 0.01; Figure 3). These findings indicate that PARP inhibitors provide significant PFS benefits regardless of whether *BRCA* mutations are located within or outside the OCCR.

## 4. Discussion

The present study investigated the PFS benefits of frontline PARP inhibitor therapy based on *BRCA1*/*2* mutation locations. Mutations within functional domains, particularly the DBD of *BRCA1* and *BRCA2*, demonstrated the most pronounced benefit, with hazard ratios of 0.34 and 0.25, respectively. In contrast, *BRCA1* mutations within the BRCT domain showed no statistically significant PFS benefit, highlighting variability in therapeutic response by mutation location.

The study is aligned with the results from the post hoc analysis of the PAOLA-1/ENGOT-ov25 trial [5,12], which explored the PFS benefits of the addition of olaparib to bevacizumab as a maintenance therapy, especially focusing on the functional domains of *BRCA* mutations. A key similarity between the two studies lies in the pronounced benefit of PARP inhibitors for patients with DBD mutations in *BRCA1*. The post hoc analysis of the PAOLA-1 trial demonstrated that *BRCA1* DBD mutations yielded the highest PFS benefit, with an impressive HR of 0.08 (95% CI, 0.02–0.28; *p* = 0.03), indicating exceptional sensitivity to the olaparib–bevacizumab combination. Our findings similarly indicate that patients with *BRCA1* or *BRCA2* DBD mutations significantly benefit from frontline PARP inhibitor maintenance therapy.

Compared to the PAOLA-1 post hoc analysis, which focused predominantly on the efficacy of the olaparib–bevacizumab combination in patients with *BRCA1*/*2* mutations, this study exclusively evaluated the frontline use of PARP inhibitors (olaparib or niraparib) without bevacizumab. Furthermore, the current analysis provides a more detailed investigation into mutation-specific outcomes within an Asian cohort, highlighting demographic and geographic variability in mutation distributions and responses.

Differences in the efficacy of PARP inhibitors across functional domains may be attributed to the presence of reversion mutation hotspots that vary by domain, potentially influencing PARP inhibitor resistance. Previous studies have proposed that, unlike other functional domains, *BRCA1 and BRCA2* DBD may be less prone to reversion mutations, potentially preserving HRD and thereby extending efficacy to PARP inhibitors [17,18,19]. Furthermore, functional domains differ in their capacity to disrupt DNA repair pathways, which directly influences synthetic lethality. *BRCA2* DBD plays a critical role in homologous recombination repair by facilitating *RAD51* recombinase activity [20]. Mutations in this domain disrupt *RAD51* loading at double-strand break sites, compromising the DNA repair process. This disruption renders tumor cells highly dependent on PARP-mediated repair pathways, making them more vulnerable to synthetic lethality induced by PARP inhibitors.

Moreover, both studies consistently demonstrated that the PFS benefit for patients with mutations in the BRCT domain of *BRCA1* is relatively modest (post hoc analysis of PAOLA-1, HR, 0.55; 95% CI, 0.2–1.56; present study, HR, 0.764, HR; 0.385–1.516). The lack of benefit may be attributed to the mechanisms underlying PARP inhibitor resistance, particularly in the *BRCA1* BRCT domain mutations. Johnson et al. demonstrated that BRCT domain mutations often lead to protein instability due to misfolding and protease-mediated degradation. However, under PARP inhibitor selection pressure, heat shock protein 90 (HSP90)-mediated stabilization of the truncated mutant protein can enable partial functionality, allowing interactions with *PALB2*-*BRCA2*-*RAD51* complexes and facilitating *RAD51* loading. This stabilization may contribute to a reduced dependency on PARP-mediated repair pathways, thereby diminishing the efficacy of PARP inhibitors [21]. In addition, Bouwman et al. highlighted that DNA repair activity persists through alternative pathways. This partial restoration of homologous recombination, facilitated by factors like TP53BP1 loss, reduces tumor dependency on PARP-mediated repair, contributing to PARP inhibitor resistance [22]. Although the smaller sample size for BRCT mutations in both studies necessitates cautious interpretation, this finding raises important questions regarding the functional implications of BRCT mutations in PARP inhibitor response.

In the current study, we further investigated the relationship between *BRCA1*/*2* mutation location and PARP inhibitor efficacy, focusing on the OCCR. The OCCR refers to regions within *BRCA1*/*2* associated with a higher risk of ovarian cancer compared to breast cancer [16]. While the OCCR classification has proven valuable for cancer risk stratification, its utility in assessing clinical outcomes and prognosis remains limited. Regarding the survival outcomes, Ha et al. reported with 162 *BRCA1* mutated patients that patients with the *BRCA1* mutation in the OCCR had a shorter PFS compared to non-OCCR in the univariable analysis [23]. However, the location of the *BRCA1* mutation in OCCR was not a significant prognostic factor for PFS, after adjusting clinical variables, including platinum sensitivity and clinical stage [23]. Our findings indicate that PARP inhibitor efficacy appears independent of OCCR status. OCCR is not strictly aligned with functional domains, requiring caution in interpreting results, as mutation effects vary based on their impact on homologous recombination and other cellular processes.

The mutation profiles in *BRCA1* and *BRCA2* exhibit notable differences between our Asian cohort and the predominantly European cohort of the PAOLA-1 trial, underscoring potential demographic and geographic variability. Notably, the PAOLA-1 trial included only 24 Japanese patients. In this study, 63.7% of patients carried *BRCA1* mutations, and 36.1% carried *BRCA2* mutations, compared to 68.2% and 31.8%, respectively, in the PAOLA-1 trial. Regarding the distribution of mutations in the functional domains, the *BRCA1* mutations in the DBD accounted for 15.4% in our cohort versus 25.2% in PAOLA-1, whereas the BRCT domain mutations comprised 21.3% and 20.8%, respectively. Similarly, *BRCA2 RAD51-BD* mutations represented 34.3% in our cohort, compared to 48.6% in PAOLA-1. These findings underscore the need to consider population-specific mutation distributions when evaluating PARP inhibitor efficacy.

This study has several limitations that should be acknowledged. First, as a retrospective analysis, it is inherently susceptible to selection bias, which may have influenced the study’s findings. Second, the results from subgroup analyses, particularly those based on specific *BRCA1*/*2* functional domains, are limited by small sample sizes within each subgroup. This necessitates cautious interpretation, as the statistical power to detect subtle differences may be compromised. Lastly, while the study identifies significant associations between *BRCA1*/*2* mutation location and PARP inhibitor efficacy, the biological mechanisms underlying such domain-specific sensitivity remain speculative.

Despite these limitations, this study utilizes a multicenter Asian cohort to provide a comprehensive analysis of both functional domains and OCCR. It builds upon the post hoc analysis of the PAOLA-1 trial by demonstrating differences in PARP inhibitor efficacy based on functional domain mutations. Additionally, the research indicates that these variations might be associated with the ability of certain domains to interfere with DNA repair mechanisms and their role in PARP inhibitor resistance processes, thus affecting treatment outcomes.

## 5. Conclusions

Frontline PARP inhibitor maintenance therapy provides a substantial PFS benefit for newly diagnosed epithelial ovarian cancer patients with *BRCA* pathogenic variants, with the most pronounced efficacy observed in mutations located within the DNA-binding domains of *BRCA1* and *BRCA2*. Conversely, the limited benefit seen in *BRCA1* BRCT domain mutations raises important questions about domain-specific therapeutic vulnerabilities.

## Figures and Tables

**Table 1:** Baseline characteristics.

	Overall N = 380	PARP Inhibitor (−) N = 171	PARP Inhibitor (+) N = 209	*p*-Value
Age at diagnosis, years				0.34 *
Median (IQR)	57 (49–64)	57 (49–64)	56 (49–63)	
Histologic type				0.01 †
High grade serous	358 (94.2%)	155 (90.6%)	203 (97.1%)	
Others	22 (5.8%)	16 (9.4%)	6 (2.9%)	
FIGO stage 2014				0.29 †
III	211 (55.5%)	100 (58.5%)	111 (53.1%)	
IV	169 (44.5%)	71 (41.5%)	98 (46.9%)	
Timing of cytoreductive surgery				0.44 ‡
Upfront	199 (52.4%)	86 (50.3%)	113 (54.1%)	
Interval	180 (47.4%)	84 (49.1%)	96 (45.9%)	
No surgery	1 (0.3%)	1 (0.6%)	0 (0%)	
Residual disease (Missing = 2)				0.53 †
No gross residual disease	243 (64.3%)	113 (66.9%)	130 (62.2%)	
Macroscopic < 1 cm	114 (30.2%)	46 (27.2%)	68 (32.5%)	
Macroscopic ≥ 1 cm	21 (5.6%)	10 (5.9%)	11 (5.3%)	
Serum CA-125 levels at initial diagnosis, IU/mL (Missing = 5)				0.20 *
Median (IQR)	1080 (381–3160)	1287 (432–3620)	972.5 (337–3023.5)	
Clinical response after platinum-based chemotherapy				
Clinical CR	341 (89.7%)	150 (87.7%)	191 (91.4%)	0.24 #
PR	35 (9.2%)	17 (9.9%)	18 (8.6%)	0.66 #
SD	1 (0.3%)	1 (0.6%)	0 (0%)	>0.99 #
Maintenance use				-
Olaparib	-	-	168 (80.4%)	
Niraparib	-	-	41 (19.6%)	

IQR, interquartile range; CR, complete response; PR, partial response; SD, stable disease. *: Wilcoxon rank sum test, †: Chi-squared test, ‡: Fisher’s exact test, #: Z-test.

**Table 2:** Mutational type and location of mutation.

	Overall N = 380	PARP Inhibitor (−) N = 171	PARP Inhibitor (+) N = 209	*p*-Value
BRCA mutation				0.52 ‡
*BRCA1*	242 (63.7%)	111 (64.9%)	131 (62.7%)	
*BRCA2*	137 (36.1%)	59 (34.5%)	78 (37.3%)	
*BRCA1* and *BRCA2*	1 (0.3%)	1 (0.6%)	0 (0%)	
Mutational type (Missing = 3)				0.16 ‡
Frameshift	175 (46.4%)	82 (48.5%)	93 (44.7%)	
Nonsense	35 (9.3%)	11 (6.5%)	24 (11.5%)	
Missense	128 (34%)	62 (36.7%)	66 (31.7%)	
Splice-site	25 (6.6%)	11 (6.5%)	14 (6.7%)	
Large rearrangement	14 (3.7%)	3 (1.8%)	11 (5.3%)	
Cluster region (Missing = 3)				0.32 †
OCCR	185 (49.1%)	87 (51.5%)	98 (47.1%)	
Non-OCCR	192 (50.9%)	82 (48.5%)	110 (52.9%)	
Specific binding domain				
*BRCA1* (N = 240)				0.22 ‡
DNA binding	37 (15.4%)	13 (11.8%)	24 (18.5%)	
DNA binding/RING	1 (0.4%)	0 (0%)	1 (0.8%)	
RING	9 (3.8%)	2 (1.8%)	7 (5.4%)	
BRCT	51 (21.3%)	26 (23.6%)	25 (19.2%)	
Others	142 (59.2%)	69 (62.7%)	73 (56.2%)	
*BRCA2* (N = 137)				0.25 †
DNA binding	42 (30.7%)	22 (37.3%)	20 (25.6%)	
*RAD51*-Binding	47 (34.3%)	20 (33.9%)	27 (34.6%)	
Others	48 (35%)	17 (28.8%)	31 (39.7%)	

OCCR, ovarian cancer cluster region; RING, really interesting gene; BRCT, C-terminal domain of *BRCA1*; †: Chi-squared test, ‡: Fisher’s exact test.

## Data Availability

The data and review protocol used in this analysis will be made available to interested parties upon request, subject to the approval of the proposed analysis plan by the investigators. To obtain access, please submit a reasonable request to the corresponding author following publication.

## References

[1] Bray F., Laversanne M., Sung H., Ferlay J., Siegel R.L., Soerjomataram I., Jemal A. (2024). Global cancer statistics 2022: GLOBOCAN estimates of incidence and mortality worldwide for 36 cancers in 185 countries. CA Cancer J. Clin..

[2] Lim M.C., Moon E.K., Shin A., Jung K.W., Won Y.J., Seo S.S., Kang S., Kim J.-W., Kim J.-Y., Park S.-Y. (2013). Incidence of cervical, endometrial, and ovarian cancer in Korea, 1999–2010. J. Gynecol. Oncol..

[3] Moore K., Colombo N., Scambia G., Kim B.G., Oaknin A., Friedlander M., Lisyanskaya A., Floquet A., Leary A., Sonke G.S. (2018). Maintenance Olaparib in Patients with Newly Diagnosed Advanced Ovarian Cancer. N. Engl. J. Med..

[4] Gonzalez-Martin A., Pothuri B., Vergote I., DePont Christensen R., Graybill W., Mirza M.R., McCormick C., Lorusso D., Hoskins P., Freyer G. (2019). Niraparib in Patients with Newly Diagnosed Advanced Ovarian Cancer. N. Engl. J. Med..

[5] Ray-Coquard I., Pautier P., Pignata S., Perol D., Gonzalez-Martin A., Berger R., Fujiwara K., Vergote I., Colombo N., Maepaa J. (2019). Olaparib plus Bevacizumab as First-Line Maintenance in Ovarian Cancer. N. Engl. J. Med..

[6] Monk B.J., Barretina-Ginesta M.P., Pothuri B., Vergote I., Graybill W., Mirza M.R., McCormick C., Lorusso D., Moore R.G., Freyer G. (2024). Niraparib first-line maintenance therapy in patients with newly diagnosed advanced ovarian cancer: Final overall survival results from the PRIMA/ENGOT-OV26/GOG-3012 trial. Ann. Oncol..

[7] Kim J.H., Kim S.I., Park E.Y., Kim E.T., Kim H., Kim S., Park S.-Y., Lim M.C. (2024). Comparison of survival outcomes between olaparib and niraparib maintenance therapy in BRCA-mutated, newly diagnosed advanced ovarian cancer. Gynecol. Oncol..

[8] Gayther S.A., Warren W., Mazoyer S., Russell P.A., Harrington P.A., Chiano M., Seal S., Hamoudi R., van Rensburg E.J., Dunning A.M. (1995). Germline mutations of the *BRCA1* gene in breast and ovarian cancer families provide evidence for a genotype-phenotype correlation. Nat. Genet..

[9] Gayther S.A., Mangion J., Russell P., Seal S., Barfoot R., Ponder B.A., Stratton M.R., Easton D. (1997). Variation of risks of breast and ovarian cancer associated with different germline mutations of the *BRCA2* gene. Nat. Genet..

[10] Krais J.J., Johnson N. (2020). *BRCA1* Mutations in Cancer: Coordinating Deficiencies in Homologous Recombination with Tumorigenesis. Cancer Res..

[11] Roy R., Chun J., Powell S.N. (2011). *BRCA1* and *BRCA2*: Different roles in a common pathway of genome protection. Nat. Rev. Cancer.

[12] Labidi-Galy S.I., Rodrigues M., Sandoval J.L., Kurtz J.E., Heitz F., Mosconi A.M., Romero I., Denison U., Nagao S., Vergote I. (2023). Association of location of *BRCA1* and *BRCA2* mutations with benefit from olaparib and bevacizumab maintenance in high-grade ovarian cancer: Phase III PAOLA-1/ENGOT-ov25 trial subgroup exploratory analysis. Ann. Oncol..

[13] Eisenhauer E.A., Therasse P., Bogaerts J., Schwartz L.H., Sargent D., Ford R., Dancey J., Arbuck S., Gwyther S., Mooney M. (2009). New response evaluation criteria in solid tumours: Revised RECIST guideline (version 1.1). Eur. J. Cancer.

[14] Millot G.A., Carvalho M.A., Caputo S.M., Vreeswijk M.P., Brown M.A., Webb M., Rouleau E., Neuhausen S.L., Hansen T.v.O., Falli A. (2012). A guide for functional analysis of *BRCA1* variants of uncertain significance. Hum. Mutat..

[15] Guidugli L., Carreira A., Caputo S.M., Ehlen A., Galli A., Monteiro A.N., Neuhausen S.L., Hansen T.v.O., Couch F.J., Vreeswijk M.P.G. (2014). Functional assays for analysis of variants of uncertain significance in *BRCA2*. Hum. Mutat..

[16] Rebbeck T.R., Mitra N., Wan F., Sinilnikova O.M., Healey S., McGuffog L., Mazoyer S., Chenevix-Trench G., Easton D.F., Antoniou A.C. (2015). Association of type and location of *BRCA1* and *BRCA2* mutations with risk of breast and ovarian cancer. JAMA.

[17] Pettitt S.J., Frankum J.R., Punta M., Lise S., Alexander J., Chen Y., Yap T.A., Haider S., Tutt A.N.J., Lord C.J. (2020). Clinical *BRCA1*/*2* Reversion Analysis Identifies Hotspot Mutations and Predicted Neoantigens Associated with Therapy Resistance. Cancer Discov..

[18] Nakamura K., Hayashi H., Kawano R., Ishikawa M., Aimono E., Mizuno T., Kuroda H., Kojima Y., Niikura N., Kawanishi A. (2024). *BRCA1*/*2* reversion mutations in a pan-cancer cohort. Cancer Sci..

[19] Tobalina L., Armenia J., Irving E., O’Connor M.J., Forment J.V. (2021). A meta-analysis of reversion mutations in BRCA genes identifies signatures of DNA end-joining repair mechanisms driving therapy resistance. Ann. Oncol..

[20] Davies A.A., Masson J.Y., McIlwraith M.J., Stasiak A.Z., Stasiak A., Venkitaraman A.R., West S.C. (2001). Role of *BRCA2* in control of the *RAD51* recombination and DNA repair protein. Mol. Cell.

[21] Johnson N., Johnson S.F., Yao W., Li Y.C., Choi Y.E., Bernhardy A.J., Wang Y., Capelletti M., Sarosiek K.A., Moreau L.A. (2013). Stabilization of mutant *BRCA1* protein confers PARP inhibitor and platinum resistance. Proc. Natl. Acad. Sci. USA.

[22] Bouwman P., Jonkers J. (2014). Molecular pathways: How can BRCA-mutated tumors become resistant to PARP inhibitors?. Clin. Cancer Res..

[23] Ha H.I., Park E.Y., Eoh K.J., Lee Y.J., Seo S.S., Kang S., Park S.-Y., Lim M.C. (2022). Clinical outcomes of *BRCA1*/*2* pathogenic variants in ovarian cancer cluster region in patients with primary peritoneal, epithelial ovarian, and fallopian tube cancer. Gynecol. Oncol..

